# Cases of Fractures

**Published:** 1856-10

**Authors:** Frank Hastings Hamilton

**Affiliations:** Buffalo, N. Y.


### ART. II.— *Cases of Fractures.* Reported by FRANK HASTINGS HAMILTON, M. D., Buffalo, N. Y.

### FRACTURES OF THE SPINE.

CASE I.—*Fracture of the transverse process of the 4th Cervical Vertebra. Death in about thirty-six hours.*

Charles Harkner, of Buffalo, aged about 40 years, was shot with a pistol, January 21, 1851. Dr. Jeyte, of Buffalo, was called. On the evening of the following day Dr. Jeyte requested me to see the case with him.

His face was blackened with powder. There was a wound on the chin, a little to the left of the center, and below the shaft of the bone, made by the bullet. He kept his head constantly turned to the right side. The left side of his neck was swollen and crepitant: left arm and leg paralyzed. He slept most of the time, unless aroused by being spoken to. He then seemed to be conscious, but was unable to speak. By signs he indicated to us that he was in no pain. He gradually sank, and died the next morning at about 6 o'clock.

Autopsy, four hours after death. We traced the wound from the chin through the left ala of the thyroid cartilage, (the wound opening into the larynx) and also through the roots of the transverse process of the 4th cervical vertebra. Immediately behind this process, embedded in the muscles, we found the bullet; it was irregularly flattened. The patient died probably from serous effusion in the spinal marrow and at the base of the brain. The emphysema under the skin of the neck was occasioned by air which escaped from the larynx through the track of the wound which has been described.

Case II.—*Fracture of the spinous process of the 6th Cervical Vertebra. Death in thirty-six hours.*

John Larkin, of Buffalo, aged about 40 years. May 1, 1851, during a violent storm of wind and rain, a ballustrade fell from a building, striking him upon the back of his head and neck. Larkin fell to the ground instantly, and never again moved his feet or legs. I saw him with Dr. H. B. Vandeventer, soon after. He was perfectly conscious, and remained so until he died. His legs and bladder were completely paralyzed. The right arm he could move pretty freely, but the left only a very little. He suffered considerable pain, especially when he was moved.

He died in thirty-six hours from the time of the receipt of the injury.

An autopsy disclosed the fact that the spinous process of the sixth vertebra was broken off at its base, and forced in upon the spinal marrow. There was no blood effused under the broken process, and we thought it might have been very easily elevated by an operation, and that some better chance might have thus been given for a final recovery.

The tunica arachnoidea, at the base of the brain, contained about one ounce of serum.

Case III.—*Fracture of the spinous process of the 7th Cervical Vertebra*

Rodney Loblin, of Hamburg, Erie county, N. Y., aged about 40 years. Temperament highly nervous. Mr. L. was thrown from a wagon in February, 1851, striking upon the back of his neck. He was insensible several hours, and when consciousness returned, his lower extremities and bladder were paralyzed. During four weeks Dr. Allen, of Aurora, drew off his water with a catheter.

Nine months after he received the injury he called upon me. I found the spinous process of the seventh vertebra pushed over to the left side. His head was strongly bent forward, and he could not straighten it. He could walk a few steps. He had almost constant pain in his lower extremities. He was restless and unable to sleep. Nearly all of the time since the accident he had taken morphine, and he was now taking it in large doses.

I recommended hot mustard baths, frictions, and a diminution in the quantity of morphine.

The following two cases, one of which I have myself seen, with the accompanying note, I shall take the liberty of publishing as reported to me by Dr. Cronyn:

"Fort Erie, Sept. 16, 1856.

"My Dear Doctor:

"I am too happy to have it in my power to furnish you any data by which much that is obscure in the diagnosis and treatment of injuries of the spine may in the least be made more clear; as you will see, I have added a few notes of another case which may not be unacceptable. What I have stated in regard to McCarty's case, might be extended very much. I have hurriedly culled from my notes of the case what you here find; I trust they may prove of some value to your paper.

"I am, very truly,
"Yours, &c.,
"Jno. Cronyn.

"F. H. Hamilton, M. D.,
"Buffalo."

Case IV.—*Probable fracture of the lower Dorsal Vertebræ, complicated with other fractures. Death after eleven months. Reported to me by the attending physician, John Cronyn, of Fort Erie, C. W.*

"Alfred McCarty, æt. 47. On October 11, 1851, while in the act of raising a piece of timber, some half a ton in weight, the elevating media gave way, the beam fell, striking McC. between the shoulders, throwing him to the left, while the timber sliding to the right side, came in contact with the knee, which being flexed and fixed at the moment, crushed the head of the tibia into pieces, driving the condyles of the femur into the upper third of its shaft, fracturing the fibula in two or three places. He was taken up insensible and powerless, and carried to the house. After the application of warmth and the administration of stimulants, he rallied sufficiently to allow a careful examination of the back: this revealed fractures of the 7th, 8th, 9th and 10th ribs of the right side at their angles, but no fracture of any of the vertebræ could be discovered. Yet from all the symptoms present it is fully believed that fractures of the vertebræ corresponding to the above ribs have existed. Motion and sensation were entirely absent from lower half of body.

"April, 1852. Up to this time McC. has steadily improved; sensation has in a great measure returned to the lower extremities, but motion only very slightly; the fracture of the ribs, tibia and fibula, partially united only; he is able to sit up provided the back is supported, and has gained flesh and strength; bedsore upon sacrum healed; catheterism, which had to be resorted to constantly at first, for the last three months, except rarely, is not needed, but as soon as the desire to urinate manifests itself, the patient has no retentive power. It is the same with the fœcal discharges. Urine highly alkaline. About this time the patient began to feel a stiffness of his hand, inability to close his fingers upon the palm, and slight uneasiness toward the cervical vertebræ. This continued to extend itself until the arms were as useless as the legs; vomiting and purging now set in, which no means permanently controlled; emaciation followed as a matter of course, little or no food being retained; whatever improvement took place for the first six months now rapidly disappeared; every evidence of the extension of inflammatory action along the cervical portions of the spinal cord was present, and after repeated attacks, in the last month of his life, of laryngo-tracheal constriction, on Sept. 20th, 1852, he died exhausted, powerless and insensible to external objects. No post-mortem examination being permitted, leaves the question of vertebral fracture in doubt.

"The treatment during the whole case consisted in simple alteratives, anodynes, tonics and counter-irritation. Dr. Hamilton, of Buffalo, saw the case in consultation with me, and suggested an expectant course, which was strictly followed."

CASE V.—*Supposed fracture of odontoid process of atlas, &c. Death in seven days.* (*Reported by Dr. Cronyn.*)

"John Mitchel, æt. 30, stone mason by trade, in Nov., 1853, being intoxicated, fell from the canal bridge, at Black Rock, a distance of about fourteen feet, upon the hard ground and upon his head or back of his neck; was picked up insensible and paralyzed. An hour after the accident I saw him. He complained only of his neck, which he could not move on account of the pain it gave him, and the poor fellow wondered much why his arms and legs would not obey his will. His breathing was entirely diaphragmatic; he complained of a choking sensation, which was much increased by an effort at deglutition. He begged to have his position changed every moment, hoping to be easier, or at least relieved from an almost incessant cough he had; his neck swelled much, as did the chest; the eyes became injected; delirium set in, and after a week's most intolerable suffering, he died convulsed. Fracture of odontoid process of dentata, and laceration of its ligaments, with displacement, were supposed to exist. A post-mortem examination would have proved the fact, but it would not be allowed. Urine had to be drawn off, and the evacuations from the bowels were involuntary. Complete paralysis of the extremities persisted from the receipt of injury."

### FRACTURES OF THE STERNUM.

CASE I. — *Simple Fracture. Recovery.*

James Connels, æt. 22, was struck by the end of the shaft of a wagon, breaking the sternum transversely near its middle. A regular surgeon was employed. He was confined to his bed six days, and suffered great pain, especially in breathing, and whenever he moved his arms. He felt the crepitus distinctly whenever he bent forward.

Five years after the accident, he was in the Buffalo Hospital, in Dr. Flint's ward, under treatment for ascites. The ascites had commenced about eight months before his admission. From the time of the accident until then, he had enjoyed good health.

I examined his sternum and found its surface quite irregular; and this irregularity, he says, was the result of the fracture. Opposite the second and third ribs, the sternum is depressed; and opposite the fourth, it is elevated.

CASE II. — *Fracture, complicated with other injuries. Immediate death* (See Case IX., of "Fractures of Ribs.")

CASE III. — *Simple Fracture. Result imperfect.*

H. B. Ruggles, æt. 28, fell forward, striking the lower end of his sternum upon the top of a candlestick, and breaking in the ensiform cartilage. For two years following the accident he had frequent attacks of vomiting, which were excessively violent. These paroxysms occurred as often as every five or six days. A great many boils formed around the epigastric region.

Dr. Green, of Albany, and Dr. White, of Cherry Valley, recommended the excision of the bone, but the patient would not consent.

In 1848, twelve years after the accident, I found the lower end of the sternum pointing directly toward the spine, but it gave him no inconvenience except that it hurt him occasionally when he coughed.

### FRACTURES OF THE RIBS.

CASE I. — *Fracture of Six upper Ribs, complicated with Fracture of the Clavicle. Result imperfect.*

C. A. W., æt. 40, fractured six upper ribs on the right side. Fracture at junction of cartilages with sternum. Owing to the severity of the bruises,

&c., no attempt was made to reduce the fragments; and indeed at first the existence of the fracture of the ribs was overlooked, and when, subsequently, the fact was noticed, it was not thought necessary, or advisable, to make any surgical interference.

The ribs have united with a very marked forward projection at the seat of fracture.

Case II.— *Comminuted fracture of the 5th rib. Emphysema. Recovery.*

Dr. Josiah Barnes, of Buffalo, æt. 50, was struck, Sept. 12, 1854, by the wheel of his carriage, and thrown down, breaking the fifth rib on the left side, about two inches from the sternal cartilage. The rib was broken by the pressure of a watch lying in the vest pocket, and Dr. B. thinks it was broken at two points.

I saw him a few minutes after the accident occurred. He was suffering greatly from the shock, and was breathing with difficulty. He complained of intense pain in his left side, in the region of his heart. I could not at this time discover crepitus, but he said he felt distinctly the motion of the fragments. Subsequently I was able to feel the crepitus.

We applied a broad bandage. He did not think this gave him much, if any, relief at the time, but subsequently it gave him great comfort. We also administered morphine, brandy, and made hot applications to the surface.

On the second day Dr. Josiah Trowbridge took charge of him, and I only saw him occasionally. About the fourth or fifth day he coughed up some blood, and a slight pneumonia was developed, which lasted a week or more; for which he was bled and took antimony. I have no doubt that the lung was penetrated by one or more of the ribs. Dr. Barnes related to me also that he felt, for a period of four months, a severe pain, with an occasional, sudden, slipping sensation, near the articulation of the last rib of the left side with the spine. He felt the motion of the broken fragments of the fifth rib about two weeks.

Sept. 9, 1856, I cannot discover any traces of the fracture, but there is occasionally pain and soreness in this region, accompanied with a cough.

Case III.—*Fracture of the 6th Rib. Recovery.*

Metz, æt. 45, was thrown down and run over by a fire engine, on the 4th of July, 1848. Dr. Mixer was called.

July 7th, being the third day after the accident, I met Dr. Mixer in consultation. The 6th rib was broken near its junction with its cartilage. The fragments were depressed. He had great pain at the seat of fracture, and considerable fever. Dr. Mixer had enclosed his body in a snug bandage, of which the patient was complaining. He had been bled and was now taking morphine and antimony.

No change was made in the treatment, and he soon recovered.

CASE IV.—*Fracture of 5th and 6th Ribs. Emphysema. Recovery.*

George Stanford, æt. 60, was thrown from his carriage, Sept. 10, 1855, breaking the 5th and 6th ribs of the left side, and at a point about six or eight inches from the sternum. When I first saw Mr. Stanford, less than two hours after the accident, crepitus was distinct. There was also a crepitus produced by the admission of air beneath the skin. It was certain, therefore, that the lungs were penetrated, yet he had no cough, nor did he expectorate blood.

I applied a broad bandage about his chest, and gave him morphine.

Sept. 13, 1856, one year after the accident, I am informed that he is perfectly well, except there is occasionally some pain in the left side.

CASE V.— *Comminuted fracture of 6th and 7th Ribs, complicated with a fracture of the Clavicle and with a wound of the Lungs. Emphysema, &c. Recovery.*

A. Vanderhyden, of Auburn, N. Y., was stabbed in his right side with a butcher's knife, the wound penetrating several inches into the right lung. The 6th and 7th ribs on the same side, were also broken by being kicked with a heavy boot. The clavicle of the same side was also broken. I saw and dressed the wound immediately after the injury was received. There was extensive emphysema of the right side—very difficult and painful respiration. The fragments of the ribs were thrown inward.

I applied a broad bandage, but without much relief. His recovery, however, was rapid and complete. The broken ribs never resumed their original positions.

CASE VI.—*Fracture of the 6th and 7th Ribs. Emphysema. Death in four hours.*

Brian Luny, æt. 4. A heavy board fell lengthwise from the top of a two story building, striking him upon his back. He was seen soon after the accident by Dr. Hackstein and myself. He survived the injury only four hours, having never rallied from the shock.

The sixth and seventh ribs, of the right side, were broken off near their spinal extremities. Crepitus and motion between the fragments were distinct. There was also an extensive emphysema over the right side. Blood was ejected both from his stomach and lungs.

CASE VII.—*Fracture of 5th, 6th and 7th Ribs. Emphysema, &c. Result imperfect.*

Wm. Dascomb, æt. 63, was struck by a locomotive, June 5, 1855, breaking three ribs, and also the acromion process of the scapula. He was also otherwise severely bruised and injured.

A few hours after the accident occurred, I found the 5th, 6th and 7th ribs, of the left side, broken about four inches from the spine. The fractures all seemed to be transverse. The spinal or outer ends of the sternal fragments were forced inward. I could not detect crepitus, but the motion of the fragments was distinct. There was at this time an extensive emphysema beneath the skin, and the side was very much discolored from bloody effusion.

He had already been bled. I directed a broad band to be applied around the chest, and that he should be allowed cooling drinks.

June 7th. The emphysema had spread over the whole of the left side of his body, so that he was enormously bloated. The features of his face could scarcely be recognized. He was allowed nourishing diet.

Sept. 2, 1856, fifteen months after the receipt of the injury, I found the left side of his chest measuring three-quarters of an inch more in diameter than the right.

I could not feel any irregularity in the union of the fractured ribs, on the back, but the muscles were too thick to enable me to determine whether such a deformity did exist or not. In front, however, the cartilages of these ribs were very much bent forward, forming a large prominence along the left side of the sternum. The functions of the chest and of the lungs did not seem to be impaired.

CASE VIII.—*Fracture of the 7th, 8th, 9th and 10th Ribs, complicated with other injuries, and death in eleven months.*

(See Case IV, of Fractures of Spine.)

CASE IX.—*Fracture of several ribs, complicated with other injuries. Emphysema. Immediate death.*

Nahun P. Thayer, of Buffalo, was killed almost instantly, Sept. 16, 1856, by the falling of a very heavy tree across his chest.

I saw him soon after the accident. I think his 5th, 6th and 7th ribs, of the left side, were broken not far from the sternum, and probably other ribs. The right clavicle was torn from its sternal articulation, and thrown backward. The sternum was broken nearly transversely just below the center: the lower fragment being forced backward. There was also extensive emphysema over the chest.

CASE X.—*Fracture of 6th and 7th Ribs. Result imperfect.*

John Burdick, of Buffalo, æt. 25, fractured the sixth and seventh ribs, on the right side, near their anterior extremities. Employed no surgeon.

Eighteen years after I found them united with a slight deformity.

CASE XI.—*Fracture of the 10th Rib. Result imperfect.*

Edward Willis, æt. 27, fractured the tenth rib, on the left side near the sternum, in July, 1851. Dr. Lansing Briggs, surgeon to the Auburn State Prison, treated the fracture.

Two months after the accident I examined the patient and found the fragments united, but the rib was bent forward at the seat of fracture.

### FRACTURES OF THE OSSA INNOMINATA.

CASE I.—*Simple fracture of the anterior superior spinous process of the Ilium. Recovery.*

John Kelly, æt. 36. Fracture of the right anterior superior spinous process of the ilium, in consequence of a fall. Admitted to the hospital, Dec. 28, 1852. Crepitus and motion distinct. Fragment displaced downward three or four lines. Hip considerably bruised.

I directed him to be laid upon his back, with his hips flexed upon his abdomen. The bone united, with the same displacement as at first, after a few days confinement.

CASE II. — *Simple fracture of the anterior superior spinous process of the Ilium. Fragment united displaced. Recovery.*

Mathias Morrison, of Buffalo, æt. 40, was crushed under a falling bank of earth, July 13, 1853. Dr. Mixer, of Buffalo, in attendance. July 14, Dr. Mixer requested me to see the case with him.

I found him lying in bed and unable to stand. There was a lacerated wound and an extensive bruise on his left hip. No shortening or eversion of the thigh. He could also slightly flex his thigh upon his body. Noticing a discoloration and swelling in the neighborhood of the anterior superior spinous process of the ilium, I pressed upon it, and felt it recede with a distinct crepitus, or a kind of click: the fragment would, however, immediately resume its place. I was able, also, to trace the line of fracture and to determine that the upper fragment included a few square inches of the crest of the pelvis.

We simply directed the patient to keep still upon his back.

CASE III. — *Simple fracture of the anterior and upper extremity of the right Ilium. Recovery.*

Joseph Joquoy, æt. 40, in the employ of the State Line R. R. Co., was caught between two cars, Feb. 10, 1854, breaking the ala of the pelvis on the right side, and producing a very severe contusion of his side and belly. The fracture had carried away the anterior and superior margin of the ilium. Crepitus and motion between the fragments were distinct.

I directed him to be laid upon his back, and the abdomen and hips to be kept covered with hot fomentations, made of hops wetted with vinegar. I also gave him morphine.

His convalescence was so rapid that I did not visit him after the third or fourth day.

CASE IV. — *Simple fracture of the anterior and upper part of the right Ilium. Fragment displaced. Recovery.*

James Roche, æt. 41, fell March 7, 1854, a height of fourteen feet, breaking off the anterior superior portion of the right ala of the pelvis. He was immediately taken to the Buffalo Hospital of the Sisters of Charity, and on the following day I found him in my ward.

The fragment was quite large, and it was displaced downward three-quarters of an inch, and forward. It was movable, and crepitus might occasionally be detected.

We did not attempt to replace the fragment, nor were any dressings applied. He was laid upon his back, with his thighs and legs moderately drawn up.

March 21, two weeks after the occurrence of the accident, he left the hospital. The fragment was at that time in the same position as at first, and movable. He was able to walk without inconvenience.

Case V.—*Fracture of the posterior superior spinous process of the Ilium. Recovery.*

Miss R., of Buffalo, æt. 16, was thrown from her horse backward, striking with her back upon the ground.

She was seen immediately by Dr. Coan, of Ovid, N. Y. Two weeks from this time she was placed under my care in Buffalo.

I found a fragment broken from the posterior superior spinous process of the ilium and displaced backward about half an inch. This displacement seemed to be produced by the action of the muscles. By pressure it could be partially, but not completely reduced.

She has suffered, heretofore, with a spinal irritation. The injured hip pains her, and the leg is sometimes numb.

I applied a compress over the loose fragment, securing it with a moderately tight bandage, and enjoined perfect rest.

She recovered completely in a few weeks.

Case VI.—*Compound, comminuted fracture of left Ilium, complicated with other fractures. Fistulous discharge four months after the accident.*

Bernard Duffie, æt. 32, was crushed, June 5, 1854, under a very heavy stone which fell upon his back. He was brought six miles to the city, and placed under my charge.

I found the left ala of the pelvis broken into several fragments; motion and crepitus were distinct. The fractures were near the superior part of the bone, commencing about two inches back of the anterior sup. spin. process, and extending backward. There was a small penetrating wound extending from a point about four inches above the ala, downward to the fracture. A small fragment of bone lay in the orifice of this wound, which I removed.

The right tibia and fibula were also broken.

I dressed the wound with a piece of lint spread with simple cerate, and directed rest and cool lotions. Having also dressed his leg temporarily, I sent him to the hospital.

Oct. 1, 1854, I found him still in the hospital. There was then a large exostosis, or mass of ensheathing callus near the seat of fracture of the ilium, and also a fistulous opening communicating with the bone.

CASE VII.—*Fracture of the rim of the Acetabulum, with displacement of the Femur. Result imperfect. Recovery.*

Michael Shuker, of Buffalo, æt. 40, was crushed under a mass of iron, weighing several tons. Drs. Loomis and Sprague in attendance. They diagnosed it as a dislocation upward and backward, (left leg,) and by the aid of six men they succeeded in reducing it, as they supposed. There was rather a grating sensation than a snap when the reduction was finally accomplished. The legs were then laid beside each other, and the knees tied together; the patient lying on his back. There seemed now no difference in the form and position of the limbs. The same gentlemen, both surgeons of experience, saw him the next day, and examined the limb, and discovered no displacement. On the night of the third day muscular spasms occurred, and on the morning of the fourth the limb was again found displaced and much swollen. On this day, the fourth, I examined the limb with Dr. Sprague. It was shortened one inch and a-half, by careful measurement. The knee was rotated inward with force; and the leg adducted. We immediately applied pulleys and soon drew the trocanter down to a point apparently opposite the acetabulum; while by measuring, we found we had made the legs of the same length. The pulleys were removed. The leg did not draw up again, nor did the foot turn in; but we had felt no sensation to indicate that the bone had slipped into its socket, nor had we felt crepitus. The legs and thighs were now laid over a double inclined plane and well secured. He remained in this condition three days more, during which Dr. Sprague saw him each day, and discovered no displacement. On the night of the seventh day the spasms returned, and in the morning the same displacement had recurred. The next day we again applied the pulleys, but we found that it would not remain in place one moment after the pulleys were loosened. Now, also, while extension was being made, I rotated the foot inward, and crepitus about the joint was very distinct.

We believed it was a case of fracture of the upper rim of the acetabulum, and desisting from all farther attempts to reduce the limb, we applied Desault's straight splint, with such moderate extension as he could bear; and this treatment was continued several weeks.

His thigh, after ten years, remains shortened about two inches, with the leg very much thrown in.

CASE VIII.—*Compound, comminuted fracture, complicated with fracture of both thighs. Death in a few hours.*

John O. Keef was crushed, Oct. 23, 1851, under a heavy stone, breaking and comminuting the pelvis on both sides, and breaking also both thighs through their necks. The femoral vein was also wounded. He was immediately carried to the Buffalo Hospital of the Sisters of Charity, and died in a few hours from the shock and loss of blood.

(To be Continued.)

